# Supplementary material for: STAT3-Mediated Promoter-Enhancer Interaction Up-Regulates Inhibitor of DNA Binding 1 (ID1) to Promote Colon Cancer Progression
Source: Int J Mol Sci. 2023 Jun 12;24(12):10041. doi: 10.3390/ijms241210041 (PMC10298663; doi:10.3390/ijms241210041)
Supplement: Supplementary file 1 [file ijms-24-10041-s001.zip › ijms-2430725-supplementary.pdf]

Supplementary figures and tables

STAT3-mediated promoter-enhancer interactions up-regulate inhibitor of DNA binding 1 (*ID1*) expression to promote colon cancer progression

Zhike Lin[1*], Ying Liu[1*], Tian Xu[1], Ting Su[1], Yingying Yang[1], Runhua Liang[1], Songgang Gu[2, 3], Jie Li[1], Xuhong Song[1], Bin Liang[1], Zhijun Leng[1], Yangsihan Li[1], Lele Meng[1], Yijing Luo[1], Xiaolan Chang[1], Dongyang Huang[1, 4#] and Lingzhu Xie[1#]

Affiliations:

[1]Department of Cell Biology and Genetics, Key Laboratory of Molecular Biology in High Cancer Incidence Coastal Chaoshan Area of Guangdong Higher Education Institutes, Shantou University Medical College, Shantou, 515041, China

[2]Department of Hepatobiliary surgery, Cancer Hospital of Shantou University Medical College, 515041, Shantou, China.

[3]Department of General Surgery, First Affiliated Hospital of Shantou University Medical College, Shantou, 515041, China

[4]Department of Central Laboratory, Cancer Hospital of Shantou University Medical College, 515041, Shantou, China

[*] These authors contributed equally to this work.

[#] Correspondence: Lingzhu Xie (lzxie@stu.edu.cn) or Dongyang Huang (huangdy@stu.edu.cn)

Supplementary figures

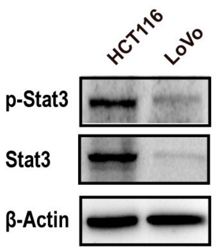

Figure S1. Expression level of STAT3 and pSTAT3 in the HCT116 and LoVo cell lines.

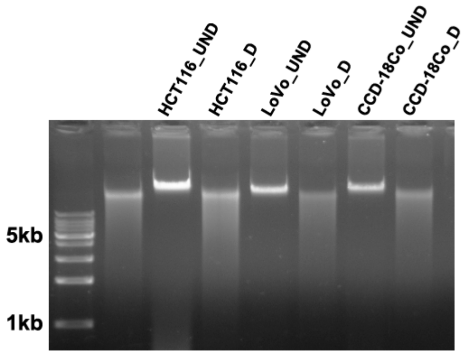

Figure S2. Efficiency of PstI digestion of the DNA from the three cell lines in the 3C assay. UND: undigested genomic DNA control, D: digested genomic DNA control.

Supplementary tables

Table S1. Histopathological features and clinical data of the patients

Case No.	Histologic type	Tumor stage	Gender †	Age (years)	Tumor stage #	Mean density *	
						normal	cancer
1	Adenocarcinoma	T4aN1aM1a	1	69	4	0.1277	0.1529
2	Adenocarcinoma	T3N0M0	2	63	2	0.0790	0.0957
3	Adenocarcinoma	T3N1aM0	1	78	3	0.0978	0.0986
4	Adenocarcinoma	T3N1aM0	1	78	3	0.1038	0.0942
5	Adenocarcinoma	T3N1aM0	1	78	3	0.1226	0.1333
6	Adenocarcinoma	T3N0M0	1	69	2	0.0576	0.0823
7	Adenocarcinoma	T3N0M0	2	53	2	0.0860	0.1988
8	Adenocarcinoma	T2N0M0	2	51	1	0.0971	0.1327
9	Adenocarcinoma	T3N1aM0	2	65	3	0.1266	0.1380
10	Adenocarcinoma	T3N0M0	1	80	2	0.0610	0.1739
11	Adenocarcinoma	T3N1aM0	1	41	3	0.1119	0.1266
12	Adenocarcinoma	T3N1bM0	2	43	3	0.1175	0.1151
13	Adenocarcinoma	T3N2aM1a	2	53	3	0.1138	0.1977
14	Adenocarcinoma	T3N1aM1a	1	73	3	0.0506	0.1238
15	Adenocarcinoma	T3N0M0	2	78	2	0.0991	0.1476
16	Adenocarcinoma	T3N0M1a	1	77	4	0.0978	0.1375
17	Adenocarcinoma	T3N0M0	2	69	2	0.0689	0.1292
18	Adenocarcinoma	T2N1aM0	2	64	3	0.0617	0.1171
19	Adenocarcinoma	T3N0M0	2	60	2	0.0629	0.1109
20	Adenocarcinoma	T3N0M0	1	65	2	0.0650	0.1079
21	Adenocarcinoma	T1N0M0	2	51	1	0.0720	0.1062
22	Adenocarcinoma	T3N1aM0	1	60	3	0.1016	0.0923
23	Adenocarcinoma	T4aN1aM1a	1	69	4	0.1155	0.1248

† Gender was assigned as follows: 1, male; 2,female.

# Tumor stage was assigned as follows:I,1; IIA-B,2; IIIA-C,3; IVA-B, 4.

* Mean density = IOD/Area (measured by IPP 6.0).The average mean density for all of five random fields at 400× magnification was used.

Table S2. Primer sequences for real-time RT- qPCR

Primer Names	Primer sequences
ID1-F	5’- TACATCAGGGACCTTCAGTTG -3’
ID1-R	5’- CTTCAGCGACACAAGATGC -3’
ID1-E1-eRNA-F	5’- GAGGAGGAGGCAAGGAATG-3’
ID1-E1-eRNA-R	5’- TTACATTCTCCTGCTCTTATTCAC -3’
ID1-E2-eRNA-F	5’- CTGGTTGACTGTTGTTCTTCC -3’
ID1-E2-eRNA-R	5’- AATTAGCCAGACTCCTCATCC -3’
ID1-E3-eRNA-F	5’- TTCACCTCCTGGGCTCTTAG -3’
ID1-E3-eRNA-R	5’- GAACCCAGGAGGCAGAGG -3’
β -actin-F	5’- TTGGCAATGAGCGGTTCC -3’
β -actin-R	5’- AGACAGCACTGTGTTGGC -3’

Table S3. Primary antibodies used for WB

antibody Names	company	Catalog Number
ID1	Santa Cruz	sc-133104
phospho-Stat3	CST	#9131
Stat3	CST	#9139
β-actin	Santa Cruz	sc-130656

Table S4. Antibodies used for ChIP

antibody Names	company	Catalog Number	dilution
normal rabbit IgG	Millipore	CS200581	3 μl/500μl
normal mouse IgG	Millipore	12-371B	3 μl/500μl
RNA polymerase II	Millipore	05-623	1 μg/500μl
H3K27ac	Thermo Fisher	720096	1 μg/500μl
H3K4me1	Abcam	ab8895	2 μl/500μl
H3K4me3	Millipore	17-614	3 μl/500μl
phospho-Stat3	CST	9131	5 μl/500μl

Table S5. Primer sequences for ChIP

Primer Names	Primer sequences
ID1-promoter-F	5'-GGCTCCGCACTCTCATTC -3'
ID1-promoter-R	5'-TGAAACAGAATGGGCAAAGC-3'
Enhancer 1-F	5'-GACCTGAGACTTGAAGAATGAG -3'
Enhancer 1-R	5'-CCCTTGCTGTTACTTGTTCC -3'
Enhancer 2-F	5'-CTGGTTGACTGTTGTTCTTCC -3'
Enhancer 2-R	5'-AATTAGCCAGACTCCTCATCC -3'
Enhancer 3-F	5'-ACAGGACGAAGCCAGGAG -3'
Enhancer 3-R	5'-AGTCCAGTGCCCAAGAAAC -3'

Table S6. Primers of sgRNA

Primer sequences for in vitro knock out of ID1.

Name	Gene ID	Sense (5'-3')	Anti-sense (5'-3')
sg-control	\	CACCGGGAGACGGGATACCGTCTCT	AAACAGAGACGGTATCCCGTCTCCC
ID1_sg4	3397	CACCGGCACGTCATCGACTACATCA	AAACTGATGTAGTCGATGACGTGCC

sgRNA sequences were acquired from high ranked hits predicted by the Feng Zhang lab (http://crispor.tefor.net/).

Primer sequences for in vitro knock out of ID1_E1.

Name	Gene Locus	sense (5'-3')	Anti-sense (5'-3')
E1_up_stream	chr20:31595376-31595395	CACCGCAGCCTCCTGCCGGGTTTGG	AAACCCAAACCCGGCAGGAGGCTGC
E1_down_stream	chr20:31597070-31597089	CACCGCCAGAGGAGGCACCCTGGA	AAACTCCAGGGTGCCTCCTCTGGC

sgRNA sequences were acquired from high ranked hits predicted by the Feng Zhang lab (http://crispor.tefor.net/).

Table S7. Primers of amplify the gRNA-directed sites

For HCT116-*ID1*_KO cells

Primer Names	Primer sequences
F_ID1_CDS	5'-ACGAGCAGCAGGTAAACG -3'
R_*ID1*_CDS	5'-CGGTATAAGGATGATCTAGTGGTC-3'

For HCT116-E1_KO cells

Primer Names	Primer sequences
test_*ID1*_sgE1_F	5'-GGCACAGCACAGAATCCTTAG -3'
test_*ID1*_sgE1_R	5'-TTCACATACCTGGCACATCTG -3'

Table S8. Plasmids and primers used in dual-luciferase reporter assays

Names	Inserted Sequences	Inserted site	Primer-F	Primer-R
pGL3-basic (basic-luc)				
promoter-luc	chr20:31604111-31605716	XhoI-HindIII	5'-CCGCTCGAGAGCGGTGAAGAAACCCCAAG -3'	5'-CCCAAGCTTTCAGCTCCAACTGAAGGTCCC -3'
promoter-luc-E1	chr20:31595068-31597104	KpnI-NheI	5'-CGGGGTACCCCCTTTGTCCAGCCACAGG -3'	5'-CTAGCTAGCCCTCCTTCTCTGCCTTCCAG -3'
promoter-luc-E2	chr20:31601917-31603926	MluI-XhoI	5'-CGACGCGTGCAGTAAACAGAATTGCTGGATC -3'	5'-CCGCTCGAGCAGCGGGCACTGTATGAAGA -3'
promoter-luc-E3-1	chr20:31606319-31608972	MluI-XhoI	5'-CGACGCGTGGGATTCCACTCGTGTGTTTC -3'	5'-CCGCTCGAGAACAGTACAGCCCCGGAAAG -3'
promoter-luc-E3-2	chr20:31607729-31610903	MluI-XhoI	5'-CGACGCGTCCTGGGCTATCGTTAGGTCC -3'	5'-CCGCTCGAGGTTGGGAACGAGGTTTTGC -3'
promoter-luc-E3-3	chr20:31610327-31611753	MluI-XhoI	5'-CGACGCGTGCGTGATCTTGGCTCATT -3'	5'-CCGCTCGAGAGGCTTGAGGAGTGCAAAGG -3'

Table S9. Primer sequences for the 3C assay

Primer Names	Paired primer	Primer sequences
Promoter1		5'- CATGGAGACGACAAACTGG -3'
Promoter2		5'- AACCAGCGGCTCAGACC -3'
PstI-1R	Promoter2	5'- CCCTTACTCTCCACACATACAC -3'
PstI-2R	Promoter2	5'- GCAGCCTTAGAGAAGTCCAC -3'
PstI-3R	Promoter2	5'- ATCTTAGGTTACTCAGCATAGGC -3'
PstI-4R	Promoter2	5'- ATTGTTGCCTTATGGTTCAGTG -3'
PstI-5R	Promoter2	5'- CTGGGAGGTGGCATTGAAC -3'
PstI-6R	Promoter2	5'- CTCTTCGTAGGGTGGTTGTG -3'
PstI-7R	Promoter2	5'- CAACCGCTTCCTGCTGAG -3'
PstI-8R	Promoter2	5'- CCCAACCCAGACCCACAG -3'
PstI-9R	Promoter2	5'- GAACACACAAGCCCAAACAG -3'
PstI-10R	Promoter1	5'- GTTAATGATTTCCAAAGGAGCAG -3'
PstI-11R	Promoter2	5'- CCAAGCAACGAACAGCAC -3'
ERCC3-1R		5'- GGTGGTAAACTCTGTCTAAAGC -3'
ERCC3-2R	ERCC3-1R	5'- TAGTTCCTGAGCCCTCCTG -3'
β-actin-3C-F*		5'- AGGAAGGAAGGCTGGAAGAG -3'
β-actin-3C-R*	β-actin-3C-F	5'- TGCGTGACATTAAGGAGAAGC -3'

*were used for loading quantity determination.

The others were for 3C-qPCR of enhancers and promoter interaction.
